# Incorporating social determinants of health into the mathematical modeling of HIV/AIDS

**DOI:** 10.1038/s41598-022-24459-0

**Published:** 2022-11-29

**Authors:** Robson Bruniera de Oliveira, Felipe Alves Rubio, Rodrigo Anderle, Mauro Sanchez, Luis Eugenio de Souza, James Macinko, Ines Dourado, Davide Rasella

**Affiliations:** 1 Institute of Collective Health, Federal University of Bahia (UFBA), Salvador, Brazil; 2 Department of Public Health, University of Brasilia, Brasilia, Brazil; 3 Departments of Health Policy and Management and Community Health Sciences, UCLA Fielding School of Public Health, Los Angeles, CA USA; 4 ISGlobal, Hospital Clínic, Universitat de Barcelona, Barcelona, Spain

**Keywords:** Infectious diseases, Epidemiology, Applied mathematics

## Abstract

Currently, it is estimated that 37.6 million people are living with the HIV/AIDS virus worldwide, placing HIV/AIDS among the ten leading causes of death, mostly among low- and lower-middle-income countries. Despite the effective intervention in the prevention and treatment, this reduction did not occur equally among populations, subpopulations and geographic regions. This difference in the occurrence of the disease is associated with the social determinants of health (SDH), which could affect the transmission and maintenance of HIV. With the recognition of the importance of SDH in HIV transmission, the development of mathematical models that incorporate these determinants could increase the accuracy and robustness of the modeling. This article aims to propose a theoretical and conceptual way of including SDH in the mathematical modeling of HIV/AIDS. The theoretical mathematical model with the Social Determinants of Health has been developed in stages. For the selection of SDH that were incorporated into the model, a narrative literature review was conducted. Secondly, we proposed an extended model in which the population (N) is divided into Susceptible (S), HIV-positive (I), Individual with AIDS (A) and individual under treatment (T). Each SDH had a different approach to embedding in the model. We performed a calibration and validation of the model. A total of 31 SDH were obtained in the review, divided into four groups: Individual Factors, Socioeconomic Factors, Social Participation, and Health Services. In the end, four determinants were selected for incorporation into the model: Education, Poverty, Use of Drugs and Alcohol abuse, and Condoms Use. the section “Numerical simulation” to simulate the influence of the poverty rate on the AIDS incidence and mortality rates. We used a Brazilian dataset of new AIDS cases and deaths, which is publicly available. We calibrated the model using a multiobjective genetic algorithm for the years 2003 to 2019. To forecast from 2020 to 2035, we assumed two lines of poverty rate representing (i) a scenario of increasing and (ii) a scenario of decreasing. To avoid overfitting, we fixed some parameters and estimated the remaining. The equations presented with the chosen SDH exemplify some approaches that we can adopt when thinking about modeling social effects on the occurrence of HIV. The model was able to capture the influence of the employment/poverty on the HIV/AIDS incidence and mortality rates, evidencing the importance of SDOH in the occurrence of diseases. The recognition of the importance of including the SDH in the modeling and studies on HIV/AIDS is evident, due to its complexity and multicausality. Models that do not take into account in their structure, will probably miss a great part of the real trends, especially in periods, as the current on, of economic crisis and strong socioeconomic changes.

## Introduction

Currently, it is estimated that 37.6 million people are living with the HIV/AIDS virus worldwide, with an associated mortality of 1.5 million people per year^1^. These numbers place HIV/AIDS among the ten leading causes of death, mostly among low- and lower-middle-income countries^2^. In 2002, the World Health Organization (WHO) implemented the getting to zero (GTZ) initiative, with the aim of making 90% of people living with HIV aware of their status, 90% of diagnosed people under adequate antiretroviral treatment (ART), and 90% of those receiving ART be virologically suppressed (with a 95–95–95 coverage by 2030)^3–7^.

Despite the effective interventions implemented for the prevention and treatment of HIV/AIDS, with a considerable reduction in new cases in the world, this reduction did not occur equally among populations, subpopulations and geographic regions^8^. This fact triggered the complexity of the challenge of controlling the disease, whose dynamics are closely related to behavioral factors, but also to social determinants to which populations are subjected^9–11^. It became clear that responding to HIV is not just a biomedical issue, but is also a social challenge.

Social determinants of health (SDH) are conditions in the environments in which people are born, live and work that influence the occurrence of health problems and their risk factors^12^. Not unlike other diseases, HIV/AIDS suffers the direct and indirect effect of these determinants, with an influence on its incidence and mortality. Socioeconomic determinants, for example, affect the dynamics of the disease, to the point that low-income populations have unequal rates in terms of HIV incidence, mortality and treatment success compared to individuals with better purchasing power^13^. Thus, low-income individuals are disproportionately affected by HIV, with an associated higher mortality^14^. Even in countries with high incomes, such as the USA, impoverished urban areas are observed to have HIV prevalence rates equivalent to those of many low-income countries with generalized epidemics^15^. In this context, a better understanding of the main social determinants of health (SDH), whether in the social, cultural and economic spheres, which interact with the risk factors for transmission and maintenance of HIV, gained space for better health outcomes and reduction of health inequities^16^.

Mathematical models, in the medical field, are used to study, among other objectives, the behavior of infectious diseases in the population, the planning and evaluation of prevention and control programs, clinical trials and cost–benefit analysis of interventions. These models can even project how infectious diseases progress over time, with the ability to predict the evolution patterns of the dynamics of a disease in populations ^17,18^. These models have been used to study the dynamics of HIV infection for decades. These models provide information about the potential impacts of interventions that are difficult to measure empirically for reasons of time, logistics or ethics^19^. With the recognition of the importance of SDH in HIV transmission, the development of mathematical models that incorporate these determinants in their scope, brings robustness to the results, with a significant improvement in the power and accuracy of the modeling^20^.

Although there are many types of models available in the literature on HIV and different statistical and mathematical approaches^21–23^, this work has its differential in terms of the proposition and development of a mathematical model of the compartmental and inclusion of social determinants. The presentation of each differential equation with the approach and way in which each determinant interacts with the other attributes of the model is the main contribution of the manuscript. The literature lacks this type of modeling, with studies with similar approaches, but focusing on another health problem, as COVID-19^20^. This article aims to propose a theoretical and conceptual compartmental model for the inclusion of social determinants of health in the structure of the mathematical models for HIV/AIDS.

## Methods

The theoretical mathematical model with the Social Determinants of Health has been developed in stages.

### Social determinants of health

**Figure 1:**
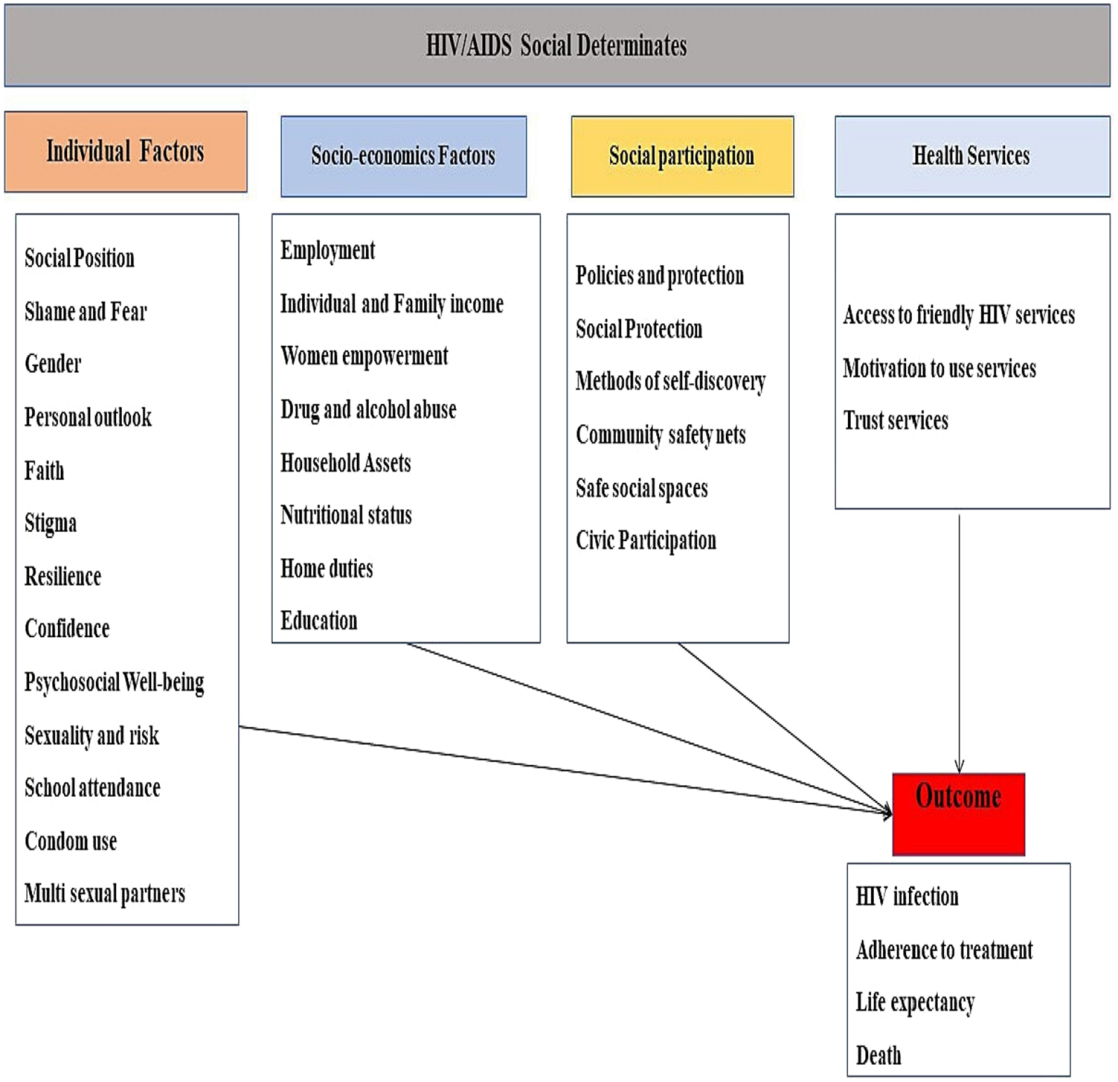
Social determinants of health for HIV/AIDS derived from narrative review.

For the selection of SDH that were incorporated into the model, a narrative literature review was conducted focused on peer-reviewed articles, in PubMed/Medline and Scopus databases between 2010 and 2020. This review included key words HIV, AIDS and Social Determinants. A total of 31 SDH were obtained in the review, divided into four groups: Individual Factors, Socioeconomic Factors, Social Participation and Health Services (Fig. 1). In the end, four determinants were selected for incorporation into the model: Education, Employment, Use of Drugs and Alcohol abuse and Condoms Use. The choice of these SDH was based on the recurrence; recognized importance in the literature and the capacity for measurement and quantification, in order to be incorporated into the equations of the Compartmental Model. Although these four chosen determinants are among the most studied and important in the field of HIV/AIDS, they do not have the capacity and robustness to represent all other determinants and groups.

### Education

Education is recognized as an important social determinant of health, being directly associated with the socioeconomic development and well-being of individuals. Different educational levels in a society lead to economic disparities and especially health inequities. Individuals with higher educational levels, in general, have better job opportunities, a lower unemployment rate, better economic conditions and, consequently, better psychosocial conditions for decision-making about their own health. Thus, education, within society, has an influence and impact on life expectancy and its morbidities^24^.

For HIV/AIDS, education plays an important role in reducing its incidence and prevalence, especially in low- and low-income countries. As many of the young people in developing countries who attend school have not yet had sexual intercourse, education for these young people is critical to the success of HIV/AIDS prevention programs. At this time, it is possible to guide young people to delay the beginning of their sexual activities, as well as encourage the use of protective methods such as condoms^25^. Petifor et al. (2016)^26^ noted that social protection programs, such as conditional cash transfer to school attendance, made young women stay in school longer, reducing the risk of acquiring HIV. Furthermore, education helps people to access and adhere to ART, as well as helping to reduce stigma, discrimination and gender inequality^27^.

### Poverty and employment

Between the years 2020 and 2021, largely as a result of the COVID-19 pandemic, the number of unemployed people in the world increased from 187.3 million to 220.3 million, the largest annual increase in unemployment in this period of time, reaching the rate average of 6.5%^28^. Many of these people who have lost their jobs have lost their only source of income, both personal and family, resulting in a drastic reduction in labor income and a consequent increase in poverty. Compared to the year 2019, 108 million more workers are now considered to be living in poverty or extreme poverty^29^. Several studies have documented the association between unemployment and poor health status^30–32^. Unemployed individuals, have their physical and mental health affected, more likely to suffer from depression, anxiety, low self-esteem, demoralization, worry and physical pain^33^. In addition, these individuals end up having their perception of health reduced, exposing themselves more to risk of HIV infection, with risk behaviors such as exchanging sex for money, often with multiple partners and without condoms; low demand for care and health services, and consequently delay in detection and initiation of treatment for HIV/AIDS and increase considerable in mortality^34,35^.

### Drug and alcohol abuse

Alcohol and drug abuse, whether injectable or non-injectable, are intrinsically associated with an increased risk of HIV infection. The main factor of injecting drugs is the sharing of syringes and needles, while alcohol and other types of drugs favor the increase in risky behavior due to the exchange of sex for drugs or money, sexual disinhibition^36^. Approximately 15.6 million people inject drugs worldwide, while around 2.8 million of those are living with the HIV virus^37^. Sharing needles and syringes has the second highest probability (of 10%) of HIV transmission per act, second only to receptive anal sex. One in 160 people becomes infected every time they share a syringe, with 10% of HIV cases in the USA being attributed to this risky behavior^38^. In this way people who inject drugs (PWID) are exposed to the double route of contamination, sexual and intravenous. Another relevant factor is that substance abuse, alcohol consumption, as well as unemployment are factors related to reduced adherence to antiretroviral treatment^39^. Therefore, the use of alcohol and drugs is a SDH that must be considered in the mathematical modeling of HIV/AIDS.

### Condom use

Approximately 95% of cases, worldwide, of people being infected by HIV are attributed to sexual practices without using condoms. Condoms are the best known, most accessible and effective method to prevent HIV infection and other sexually transmitted infections, such as syphilis, gonorrhea and also some types of hepatitis^40^. Consistent condom use can reduce HIV transmission among serodiscordant individuals by up to 80%^41^.

### Mathematical modeling

**Figure 2:**
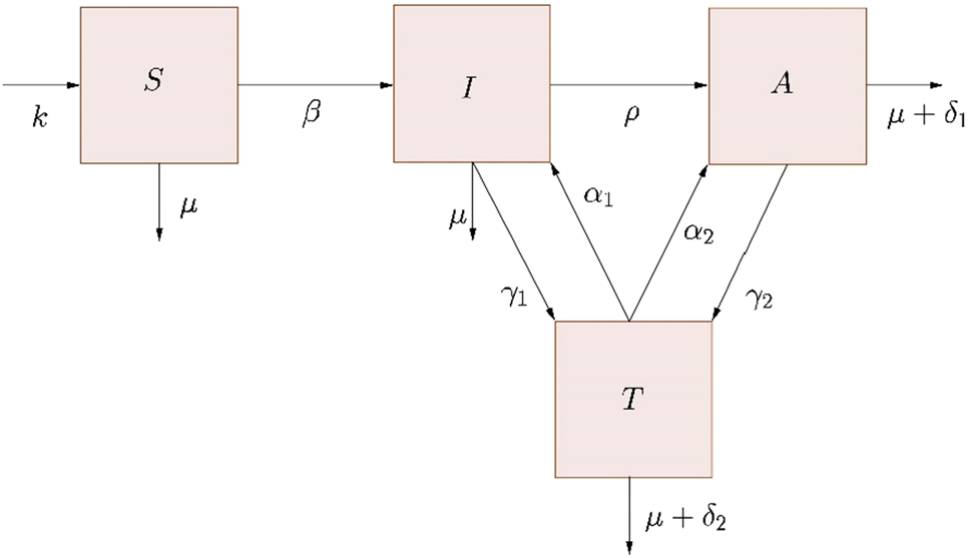
Epidemiological scheme of the HIV/AIDS transmission model.

Compartmental model is a type of mathematical model that simulates the disease status of individuals within populations, which are divided into different compartments. Within each compartment, people are considered homogeneous in terms of their behavior and risk factors^16,17^. The most generic and widely used model is the SIR model, in which individuals are classified into three types of compartments: Susceptible (S), Infected (I) and Recovered (R). Susceptible individuals are those who have never had the disease or are likely to become infected. After infection, these individuals migrate to the infected compartment and can spread the disease to susceptible individuals. The recovered ones can develop lifetime immunity or return to the Susceptible compartment, depending on the pathophysiology of the disease. In the case of HIV, the Recovered compartment does not exist, as this disease has no cure. However, a variation of the SIR model for HIV is the replacement of the Recovered compartment by the virally suppressed compartment (T), where individuals in these compartments have an undetectable viral load and their contribution to transmission is almost zero^42^. For this study, we propose an extended model in which the population (N) is divided into Susceptible (S), HIV-positive (I), Individual with AIDS (A) and individual virally suppressed (T). Thus, we consider the total population (N), where N = S + I + A + T. One way to model the dynamics of virus transmission is given by the system of equations:

$$\frac{dS}{dt}= \kappa - \mu S - \beta \frac{I}{N}S$$

$$\frac{dI}{dt}= \beta \frac{I}{N}S{+\alpha }_{1}T-\rho I -{\gamma }_{1}I-\mu I$$

$$\frac{dA}{dt}= \rho I {+\alpha }_{2}T- {\gamma }_{2}A -{\delta }_{1}A-\mu A$$

$$\frac{dT}{dt}= {\gamma }_{1}I+{\gamma }_{2}A -{\delta }_{2}T-{\alpha }_{1}T-{\alpha }_{2}T-\mu T$$

where $\kappa$ represents the population's natality rate and $\mu$ the natural mortality rate (inverse of the average life expectancy). $\beta$ represents the effective contact rate, I/N the fraction of infected individuals. Thus, the term $\beta \frac{I}{N}$ represents the HIV transmission rate. After a period ${\rho }^{-1}$, the infected individual becomes “full-blown aids”. We consider that the individual in class I, with the assumption that treatment occurs, after a time ${{\gamma }_{1}}^{-1}$ they reaches undetectable viral load levels, the same applies to the individual in compartment A, in this case after a time ${{\gamma }_{2}}^{-1}$. However, if this individual with an undetectable viral load ceases treatment, the viral load may increase, which may lead the individual to class I (infectious) or even to compartment A, with rates ${\alpha }_{1}\mathrm{and}$ ${\alpha }_{2}$, respectively. We assumed that individuals in compartment A have HIV-related death, ${\delta }_{1}$, as well as for individuals in compartment T, ${\delta }_{2}$ (with ${\delta }_{2}<{\delta }_{1}$). Figure 2 shows the epidemiological scheme of the HIV/AIDS transmission model.

### Education

One way to include the education factor would be to incorporate a new compartment, denoted here by (R-removed), representing individuals who through educational campaigns change their sexual behavior so as not to be susceptible to HIV transmission, as shown by^42^. Parameter ${\theta }_{1}$ represents this behavior. However, these individuals can change their behaviors again and return to infection susceptibility at a rate ${\theta }_{2}$. The dynamics of this class is given by the equation,

$$\frac{dR}{dt}={ \theta }_{1}S-{ \theta }_{2}R-\mu R$$

Bearing in mind that as we incorporated a new compartment into the model, the total population is now given by N = S + I + A + T + R and the susceptible dynamics is modified by including the term ${-\theta }_{1}S$.

### Condom use

To incorporate the effect of condom use, we can include this factor through the parameters $\varepsilon$ and $\nu$, which represent the condom efficacy and compliance, respectively. So, the product $c=\varepsilon \nu$ represents the level of protection against HIV through condom use^43^. We modified the effective contact rate, which goes as follows:

$$\beta =(1-c)\beta $$

where $1-c$ measures the failure to prevent transmission through condom.

### Drug and alcohol abuse

One way to incorporate the drug abuse as SDH in a mathematical model is to restrict the study to this subpopulation, that is, consider a compartmental model in which all compartments are related to drug users^44^. For instance, Burattini et al. (1998)^45^ showed the impact of crack-cocaine use on the prevalence of HIV/AIDS among drug users. Alcohol abuse can be incorporated in the model decreasing (or increasing) the parameter values, which can be influenced by alcohol use. For instance, the level of protection against HIV through condom use ($c$) as

$$c={\phi }_{c}c$$

with ${\phi }_{c}<1$, representing the alcohol abuse effect. In the case of absence of alcohol abuse effect in the mathematical model, we assume ${\phi }_{c}=1.$

### Poverty and unemployment

The inclusion of factors such as poverty and unemployment can be applied to several parameters in the model. Galanis and Hanieh (2021)^19^ explore the use of these factors by modifying the transmission rate by a linear approximation of

$$\beta {(x}_{1},{x}_{2}) = {\beta }_{0}+{\beta }_{1}{x}_{1}+{\beta }_{2}{x}_{2}$$

where ${x}_{1}$ and ${x}_{2}$ are the poverty and unemployment rates, respectively.

We can apply this modification to parameters such as ${\alpha }_{1}$, ${\alpha }_{2}$, ${\gamma }_{1}$ and ${\gamma }_{2}$ , as they can also be influenced by social determinants. Thus, the model with the inclusion of the social determinants mentioned here has the following form:

$$\frac{dS}{dt}= \kappa - \mu S - (1-c)\beta {(x}_{1},{x}_{2}) \frac{I}{N}S{ -\theta }_{1}S+{ \theta }_{2}R$$

$$\frac{dI}{dt}=(1-c)\beta {(x}_{1},{x}_{2}) \frac{I}{N}S{+\alpha }_{1}T-\rho I -{\gamma }_{1}I-\mu I$$

$$\frac{dA}{dt}= \rho I {+\alpha }_{2}T- {\gamma }_{2}A -{\delta }_{1}A-\mu A$$

$$\frac{dT}{dt}= {\gamma }_{1}I+{\gamma }_{2}A -{\delta }_{2}T-{\alpha }_{1}T-{\alpha }_{2}T-\mu T$$

$$\frac{dR}{dt}={ \theta }_{1}S-{ \theta }_{2}R-\mu R$$

### Numerical simulation

Here, we present an application of the model described above. We fitted the model with data of new cases and deaths of AIDS collected from the Brazilian Health Ministry^46^. We estimated the natural mortality rate and birth rate in order to the model approximate the total population of Brazil between 2003 and 2030. Table 1 shows the values of parameters used in the simulations.

**Table 1:** Parameter description of the HIV/AIDS model.

Parameter	Description	Value	References
$\upkappa$	Birth rate	4,590,490.56	^47^
$\upmu$	Natural mortality rate	1/75.50	^47^
${\uptheta }_{1}$	Rate of susceptible individuals who changed their habits	0.03	^42^
${\uptheta }_{2}$	Rate of susceptible individuals who changed their habits and return to the susceptible compartment	0.003	^42^
$\upbeta$	HIV transmission rate	(0, 10)	Assumed
$\upvarepsilon$	Condom efficacy	(0.96, 0.99)	Assumed
$\upnu$	Prevalence of consistent condom use	0.228	^48^
$\uprho$	Progression rate to A from I	(1/15, 1/10)	^2^
${\upgamma }_{1}$	Progression rate to T from I	2	^49^
${\mathrm{\alpha }}_{1}$	Treatment failure rate (T to I compartmental)	(0, 0.4)	^50,51^
${\mathrm{\alpha }}_{2}$	Treatment failure rate (T to A compartmental)	(0, 0.4)	^50,51^
${\updelta }_{1}$	AIDS-related death rate (individuals with full-blown aids)	(0, 0.1)	^52^
${\updelta }_{2}$	AIDS-related death rate for individuals being treated	0.0667	^52^
${\upgamma }_{2}$	Progression rate to T from A	(0.2, 2)	^38,49^

**Figure 3:**
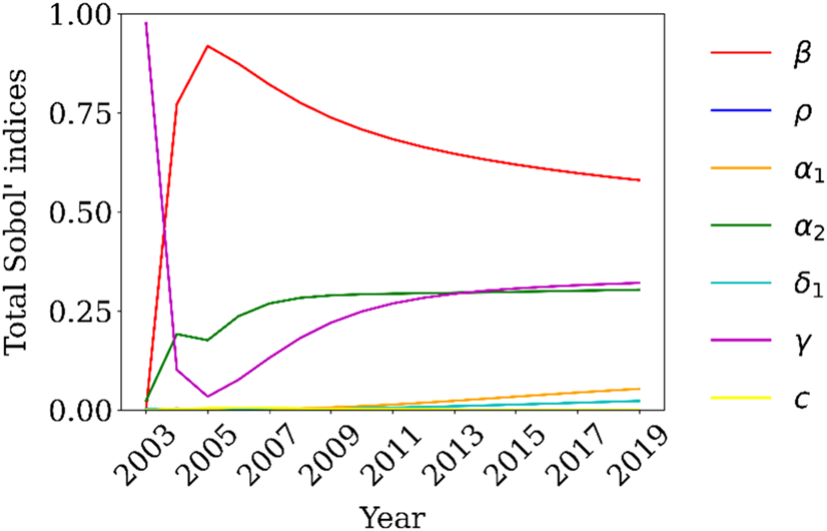
Total Sobol’ indices for the A compartment.

Assuming the range for each parameter shown in Table 1, we conducted a sensitivity analysis using a statistical variance-based method^53^ to evaluate the model parameter effects in the dynamics of the all variables over time. Since we considered AIDS data for the calibration process, we present in Fig. 3 the sensitivity analysis for the A compartment over 2003 to 2019. The most influent parameter, for the AIDS compartment, is the HIV transmission rate.

**Figure 4:**
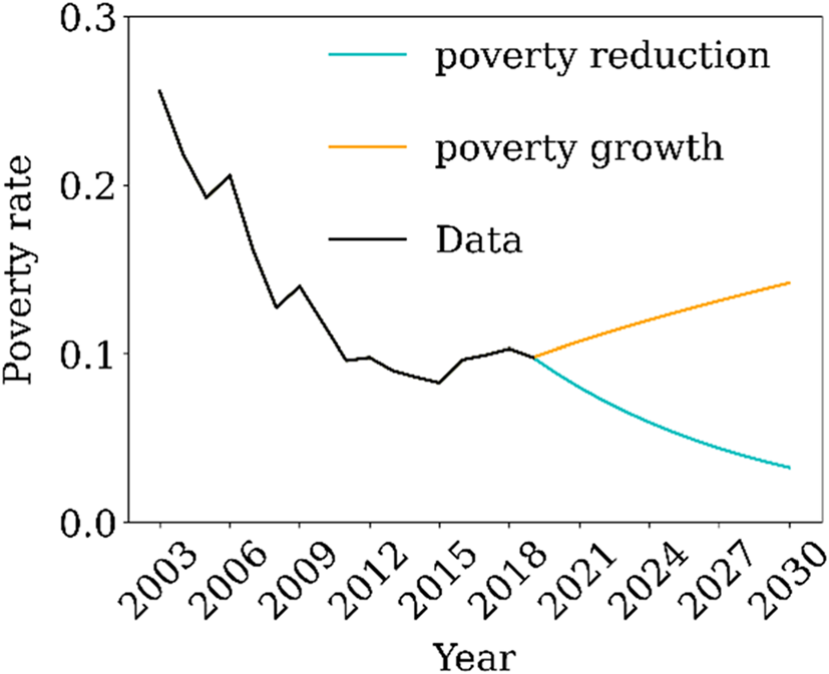
Poverty rate scenarios between 2003 and 2030.

Hence, we assumed the correlation of poverty for only this parameter. The poverty rate was calculated using three National Household Surveys (PNAD)—the regular PNAD, Continuous-PNAD, and PNAD-COVID for 2001–2011, 2012–2019, and 2020, respectively. After 2020, we assumed two different scenarios of poverty rate, scenarios of growth and poverty reduction between 2020 and 2030 (Fig. 4). We forecast the HIV/AIDS incidence and mortality rates (per 100,000 individuals), according to the level of poverty (Fig. 4).

**Figure 5:**
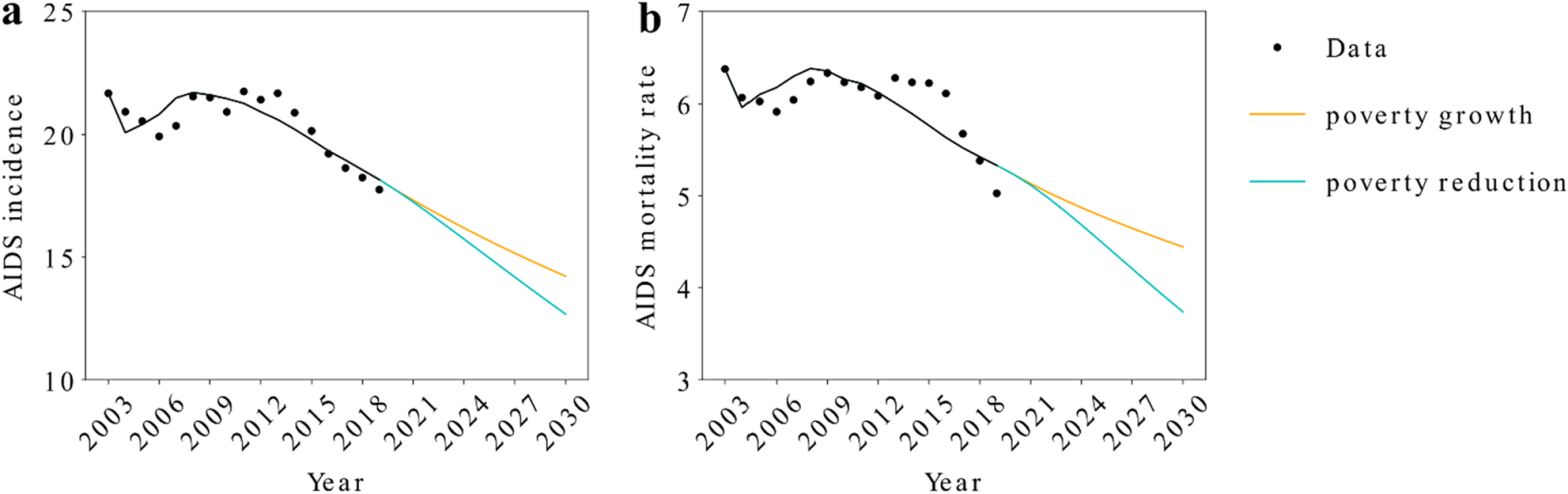
HIV/AIDS incidence and mortality rate under different scenarios of poverty between 2003 and 2030.

Figure 5 shows that depending on the level of poverty in the country, in the near future, we can expect of an increase (decrease) on these health outcomes. This result shows the importance of governmental policies to poverty mitigation in order to reduce the incidence and mortality of HIV/AIDS.

## Discussion

Although many advances have occurred in recent decades in the detection, treatment and prevention of HIV/AIDS in the world, there are still a large number of people at risk of infection or living with HIV without access to care and treatment^54,55^. The positive progress in controlling the disease, caused a drop in the number of deaths by 64% since 2010, going from 1.9 million in 2004 to 680,000 people in 2020. However, this progress is uneven within and between countries, varying widely with age, gender and especially economic status of people^1^. Thus, the African continent, being the poorest, remains the most severely affected, with almost 1 in every 25 adults (3.6%) living with HIV. The east of the African continent, one of the poorest regions in the world, even comprising only 6.2% of the world population, reports the highest numbers of HIV/AIDS cases in the world, with a prevalence of 54% of the population^56^. It is clear that not only biomedical measures are sufficient for the control and total elimination of HIV/AIDS, but the understanding and incorporation of social, cultural and environmental factors play important roles in the dynamics of the disease^57^.

Recognition of the importance of social determinants of health in the distribution and impact of a disease on a given population is a consolidated concept in the literature. Thus, the circumstances in which individuals grow, live and age play as important a role as biological factors in the occurrence of diseases^58,59^. The incorporation of these determinants in the design of epidemiological studies, with the development of new methodologies and the adaptation of methods that are already well used, to adapt to the new conditions, is a challenge that researchers have faced. The model was able to capture the influence of the employment/poverty on the HIV/AIDS incidence and mortality rates, evidencing the importance of SDOH in the occurrence of diseases.

This article aimed to propose a compartmental mathematical model for HIV/AIDS that includes SDH in its structure. The equations presented with the chosen SDH exemplify some approaches that we can adopt when thinking about modeling social effects on the occurrence of HIV. The role of mathematical modeling, in this context, is to support the foundation of the debate on the impact that a certain action or implemented program can have on a population, without losing the effect and influence that social and environmental factors play to the point of compromising the success of a well-planned program^60^. Deepening the understanding of current SDH and the search for identification of new factors, as well as a full understanding of the interaction mechanisms, is something that must always be present when conducting studies and interpreting the results. The more factors are added to mathematical models, the more robust and capable of approximating reality they become.

This article has limitations. The structure of the model chosen for this study has a simple structure with few factors. The inclusion of many factors at the same time in the model results in greater complexity, bringing with it a greater need for data to carry out its validation and calibration, which often may not be available. In addition, too many variables in the model at the same time can mask the influence of a key parameter of interest. The inclusion of only 4 SDH of the 31 identified by the literature review can be considered another limitation However, its innovation lies in the proposition of a new methodological approach and in the development of a model that includes SDH in its differential equations, working as framework and starting point for similar studies. Also. these SDOH have been selected based on their strongest effect on HIV/AIDS dynamics.

## Conclusion

The recognition of the importance of including the SDH in the modeling and studies on HIV/AID is evident, due to its complexity and multicausality. Models that do not take into account SDH in their structure, will probably miss a great part of the real trends, especially in periods, as the current on, of strong socioeconomic changes and economic crisis.

## Data Availability

The datasets generated during and/or analysed during the current study are available in the Boletim Epidemiológico HIV/Aids 2021, Ministry of Health, Brazil.repository, http://www.aids.gov.br/pt-br/pub/2021/boletim-epidemiologico-hivaids-2021. All data are within the manuscript and additional files.

## Abbreviations

GTZ: Getting to zero
PWID: People who inject drugs
SDH: Social determinants of health
WHO: World Health Organization
